# Resolving Key Barriers to Advancing Mental Health Equity in Rural Communities Using Digital Mental Health Interventions

**DOI:** 10.1001/jamahealthforum.2021.1149

**Published:** 2021-06-04

**Authors:** Andrea K. Graham, Ruth Striegel Weissman, David C. Mohr

**Affiliations:** Center for Behavioral Intervention Technologies, Northwestern University Feinberg School of Medicine, Chicago, Illinois (Graham, Mohr); Department of Psychology, Wesleyan University, Middletown, Connecticut (Weissman)

In the United States, mental health problems and risk for suicide are inequitably higher among individuals living in rural compared with urban communities.^1^ This problem is complicated by numerous barriers to adequate care that affect rural communities.^2^ Individuals living in rural areas face stigma and have concerns about disclosing mental health problems or being seen by their neighbors when accessing mental health services.^1,2^ A culture of self-reliance also makes it less likely for individuals to seek mental health services.^1,2^ For those who do want services, many live in federally designated mental health professional shortage areas, meaning mental health clinicians are scarce. Access to available clinicians may require traveling long distances, making attendance at sessions more challenging.

Digital mental health interventions (DMHIs), which use web and mobile applications to support patients and clinicians through the remote delivery of mental health services to manage mental disorders,^3^ offer a potential solution to address these barriers and increase access to mental health care, and may be particularly acceptable and beneficial for individuals living in rural areas. Given their efficacy and growing evidence of cost-effectiveness,^3^ DMHIs can expand the capacity and reach of health care settings to manage mental health. With proper privacy policies, DMHIs can provide anonymity in the use of services, which can reduce concerns around stigma and disclosure and promote engagement.^4^ The availability of DMHIs outside brick-and-mortar settings reduces barriers associated with accessing services. Although fewer rural adults own smartphones, tablets, and/or computers compared with urban and suburban residents, rural residents’ technology adoption and use has steadily risen consistent with their counterparts.^5^ These factors make digital delivery a potentially critical modality by which to scale up mental health services to many rural individuals in critical need of care.

Efforts are underway to facilitate broad adoption and integration of DMHIs into the US health care system, including through establishing reimbursement mechanisms to support their delivery.^3^ Such efforts have the potential to help ameliorate inequities in access to mental health services that exist across geographic locations, and align with broader national imperatives to reduce health inequities for rural populations.^6^ However, effectively ameliorating such inequities requires an understanding of certain affordances of DMHIs that could put rural communities at a distinct disadvantage for being able to reap the full benefits of these services. Therefore, solutions to broadly integrate DMHIs into the US health care system must consider solutions that adequately account for and address these potential barriers.

## Fitting Mental Health Services Into the Fabric of People’s Lives

A key innovation of DMHIs is their ability to fit into the fabric of people’s lives so that mental health services are available and can be used in the moments and contexts when needed most. This novel aspect of DMHIs has disrupted the standard face-to-face or telehealth-delivered psychotherapy model, in which mental health services typically are delivered synchronously in weekly sessions. With DMHIs, services can be accessed at any time based on individuals’ needs.

Yet, it is well established that rural communities lack consistent internet and/or cellular coverage.^2,5^ Because many DMHIs rely on connectivity for the patient’s phone or device, rural individuals may be disadvantaged to use DMHIs in the moments when needed most. Ensuring that DMHIs are an accessible intervention option for all requires continued attention to expanding connectivity. Federal, state, and private sector initiatives are making progress toward this goal, such as through the Coronavirus Aid, Relief, and Economic Security (CARES) Act^7^ or public-private partnerships led by the Federal Communications Commission and the National Cancer Institute. However, policies to support universal coverage are still needed. In the meantime, increased accessibility of these interventions may be helped by health care delivery innovations such as providing digital subsidies to use DMHIs^8^ and expanding community partnerships that provide private spaces to access digital tools. Companies and researchers creating DMHIs also can be attentive to building tools with core functionality that can operate without consistent connectivity.

## Facilitating Seamless Remote Patient Monitoring Through Interoperability

Another innovation of DMHIs is their ability to monitor patients remotely and transmit data to other digital platforms (eg, electronic health records [EHRs]) to inform clinical decision-making and facilitate measurement-based care. This affordance can reduce the need for in-person health care visits, which may be particularly important in rural settings, as travel distances and lack of public transportation can be prohibitive to seeking care.

As reimbursement efforts for DMHIs are pursued, the expense required to facilitate interoperability cannot be undervalued, and reimbursement payments should incentivize underserved areas to implement interoperability by accounting for differences in resources. Practices in underserved areas, such as rural-based health care practices, have been slower to adopt EHR systems. Further, rural practices that do have an EHR system use less of the health information technology functionality of the EHR compared with their metropolitan counterparts^9^; they also may have less capacity relative to larger health care systems to allocate resources to build, test, and maintain custom fields within their EHRs for DMHI data. Therefore, support for DMHI delivery will be reinforced by policies that promote EHR adoption. Additionally, equity in mental health service delivery could be bolstered by reimbursement policies for DMHIs that offer higher incentives to underserved areas to support the expense of integrating DMHI data into the EHR. Moreover, regardless of location, large-scale EHR systems can contribute to equity in health care by enabling easy integration with external digital platforms such as DMHIs, and DMHIs could improve integration by using standardized, validated metrics such as symptom monitoring, medication adherence and adverse effects, or other factors related to managing the care of patients.

## Reducing Reliance on Licensed Specialty Clinicians

The third key innovation of DMHIs is reduced reliance on licensed specialty clinicians for mental health care delivery. Digital mental health interventions are no less efficacious when supported by non-mental health personnel (eg, those with no health care or counseling qualifications) than mental health specialists.^10^ Because rural areas face known challenges with having an adequate network of specialty mental health clinicians, the ability to task-shift makes DMHIs a welcome solution to expand access to mental health services.

However, to fully realize the benefit of this affordance of DMHIs for rural areas, policy efforts should ensure that billing codes are not limited to only mental health specialists, and that such codes allow non-mental health personnel to deliver DMHIs. Reimbursement models also would benefit from compacts that enable individuals trained to deliver DMHIs to do so without geographic boundary distinctions, meaning relaxing state licensing requirements that specify that services can only be delivered within the state in which the individual is licensed. Such restrictions are problematic for rural areas that do not have enough clinicians to deliver services or for organizations that serve people across state lines. Such policy changes will ensure that DMHIs are accessible across geographic locations and that rural areas are not penalized for having insufficient numbers of clinicians to deliver these services.

## Conclusions

Digital mental health interventions may be particularly beneficial for addressing mental health care inequities in rural communities. However, certain affordances of DMHIs could inadvertently widen disparities in treatment access. With efforts under way to facilitate the broad integration of DMHIs into the US health care system, careful attention is needed for solutions that address these potential barriers for DMHI delivery in rural settings. Doing so will ensure a more equitable integration of DMHIs into the US health care system.
